# Effects of Oral Administration of Bamboo (*Dendrocalamus membranaceus*) Leaf Flavonoids on the Antioxidant Capacity, Caecal Microbiota, and Serum Metabolome of *Gallus gallus domesticus*

**DOI:** 10.3389/fnut.2022.848532

**Published:** 2022-03-03

**Authors:** Guangtian Cao, Yang Yu, Huixian Wang, Jinsong Liu, Xiping Zhang, Yue Yu, Zhanming Li, Yan Zhang, Caimei Yang

**Affiliations:** ^1^College of Standardisation, China Jiliang University, Hangzhou, China; ^2^Key Laboratory of Applied Technology on Green-Eco-Healthy Animal Husbandry of Zhejiang Province, Zhejiang Provincial Engineering Laboratory for Animal Health and Internet Technology, College of Animal Science and Technology, Zhejiang A & F University, Hangzhou, China; ^3^Zhejiang Vegamax Biotechnology Co., Ltd, Anji, China; ^4^School of Grain Science and Technology, Jiangsu University of Science and Technology, Zhenjiang, China

**Keywords:** bamboo leaf flavonoids, antioxidant capacity, caecal microbiome, serum metabolome, *Gallus gallus domesticus*, broiler feed gain

## Abstract

The consumption of bamboo leaf flavonoids (BLFs) as novel dietary antioxidants has increased owing to their beneficial biological and pharmacological functions. This study assessed the *in vivo* effects of BLFs on antioxidant capacity, as well as caecal microbiota, serum metabolome, and health status. The *Gallus gallus domesticus* model and the oral administration approach were used with four treatment groups (basal diet, basal diet with 20 mg bacitracin/kg, basal diet with 50 mg BLF/kg, and basal diet with 250 mg BLF/kg). Ultra-high-performance liquid chromatography triple-quadrupole mass spectrometry analysis indicated that vitexin, fumaric acid, orientin, isoorientin, and *p*-coumaric acid were the predominant BLF components. From days 1 to 21, BLF increased the average daily gain and decreased the feed:gain of broilers. Moreover, BLF enhanced the serum antioxidant capacity and immune responses. Further, 16S rRNA sequencing showed that BLF modulated the caecal microbial community structure, which was dominated by Betaproteobacteriales, *Erysipelatoclostridium*, *Parasutterella*, *Lewinella*, *Lactobacillus*, and *Candidatus Stoquefichus* in BLF broilers. Among the 22 identified serum metabolites in BLF broilers, sphinganine, indole-3-acetaldehyde retinol, choline, 4-methylthio-2-oxobutanoic acid, and *L*-phenylalanine were recognised as biomarkers. In summary, BLFs appeared to modulate the caecal microbiome, alter the serum metabolome, and indirectly improve antioxidant capacity and health status.

## Introduction

Nowadays, natural antioxidant compounds’ demand is huge, which synthetic antioxidants have been widely used in food and pharmaceutical industry and are likely harmful to health (1). The key roles of antioxidants are to improve cardiovascular health, inhibit the proliferation of cancerous tumours, delay the progression of brain ageing, and reduce the potential damage of neurodegenerative sicknesses (2). Natural flavonols, flavones, flavanones, and other classes of compounds have strong antioxidant and anti-inflammatory capacities (3, 4). Dietary flavonoids are common secondary metabolites detected in a plethora of vegetables and medicinal plants, which exhibit a huge number of beneficial roles and have a crucial effect on preventing chronic and degenerative sicknesses (5).

Bamboo is found worldwidely, and its leaves are applied to medicinal and culinary roles in China (6). Different parts of bamboo, such as leaves and shoots, have enormous therapeutic potential capacities, and might provide a natural and eco-friendly method in protecting health in a sustainable method, although bamboo has been rarely studied for its antioxidant capacity (1). Accumulating evidence suggests that bamboo leaf extract (BLE) consists of active substances, for instance bamboo leaf flavonoids (BLF), polyphenols, and polysaccharides, among which BLFs are the major active components (4). BLE antioxidants are complex mixtures of natural antioxidant substances that have been listed in the Chinese National Standard GB 30615-2014 and that were reported to possess strong antioxidant activity and inhibit the free radical-induced deterioration of macromolecules *in vitro* (7). It has previously been revealed that the main antioxidant components in bamboo leaves are flavonoids, lactones, and phenolic acids. Additionally, the water-soluble antioxidants of bamboo leaves are nutritional antioxidant substances and have been considered potential flavone C-glycoside-rich food antioxidants (8, 9).

Bamboo leaf extract rich in phytochemicals and antioxidants have also been used to stimulate immune response and reduce the risk of age-related chronic diseases (10). Typically, beneficial polyphenols are essential antioxidants and have outstanding antibacterial effects (11). An increase in the antioxidant capacity of hyperlipidaemic mice was reported after they were supplemented with BLE (12). It was also found that BLE supplementation significantly improved the growth performance and antioxidant capacity of birds and alleviated oxidative stress by increasing the contents of antioxidant in serum and liver (6). Flavonoids have remarkable antioxidant activity owing to their reactions with free radicals, in which they serve as hydrogen donors (13). Therefore, it is reasonable to suspect that BLFs play a major part in improving antioxidant enzyme activities (6). Furthermore, a study conducted by (14) confirmed that dietary plant-derived flavonoid supplements play an important role in reshaping the intestinal microflora and provide hosts with beneficial effects, such as immunoenhancement (14). The present study was aimed to investigate the effects of BLF on the health status, antioxidant capacity, immune response, caecal microbial community, and serum metabolome, *in vivo*, in the early growth stages of *Gallus gallus domesticus*.

## Materials and Methods

### Chemicals and Regents

For the chemical analysis, standards of caffeic acid, orientin, isoorientin, *p*-coumaric acid, vitexin, fumaric acid, and chlorogenic acid were bought from Sigma-Aldrich (St. Louis, MO, United States), which both had purity above 99.9%. Acetonitrile, methanol, and formic acid were purchased from Merck KGaA (Darmstadt, Germany). And, the Millipore Milli-Q water purification system (Bedford, MA, United States) was used for the purification of ultrapure water.

### BLF Substance Characterisation

The bamboo (*Dendrocalamus membranaceus*) leaf flavonoids were provided by Zhejiang Vegamax Biotechnology Co., Ltd (Anji, China) and consisted of 24.2% flavonoids, 12.5% lactones, and 15.6% phenolic acids. Ultra-high-performance liquid chromatography (UHPLC) analysis was conducted on the Agilent 1290 HPLC system (Agilent Technologies, Santa Clara, CA, United States) with an auto-sampler, online degasser, and a quaternary pump. An Agilent Poroshell 120 column (3.0 × 100 mm i.d.; 2.7 μm) was used for separating flavonoid substances. The mobile phase A was water consisting of 0.1% formic acid (v/v), while phase B was acetonitrile consisting of 0.1% formic acid (v/v), which the flow rate was 0.25 mL min^–1^. The linear gradient elution program was: 0–2 min, 1% B; 2–10 min, 10% B; 10–18 min, 45% B; 18–40 min, 95% B; 40–50 min, 95% B; 50–55 min, 1% B; 55–70 min, 1% B. And, the analyte injection volume was 2 μL, and the column temperature was 30°C.

The mass spectrometry (MS) analysis was carried out by the Agilent 6495 Triple Quadrupole Mass Spectrometer (Agilent Technologies). Multiple reaction monitoring (MRM) in negative ion mode was used. The MS conditions were: 3.5 kV capillary voltage, 380 V fragmentor voltage, 290°C drying gas temperature, 350°C sheath gas temperature, 12 L min^–1^ flow rate and 275.79 kPa nebulizer pressure. The precursor and product ions and their corresponding optimized collision energy values for seven flavonoid compounds are shown in Table 1.

**TABLE 1:** Optimum precursor/product ions, retention time and their corresponding collision energy values, and concentrations for seven flavonoids in BLF.^1^

No.	Identified flavone compounds	RT (min)	Precursor ion (m/z)	Production (m/z)	Collision energy (V)	Regression equation y = ax + b	R^2^	Concentration ranges (μ g mL^–1^)	Concentration (mg/100 g) Mean ± SD
1	chlorogenic acid	2.837	353.0	190.9*	20	y = 483.7x - 281.8	0.998	0–40	21.33 ± 1.12
2	caffeic acid	4.282	179.0	135.1*	20	y = 1757.8x + 77.8	0.999	0–40	1.85 ± 0.07
3	orientin	5.526	447.1	327.2*	33	y = 545.5x - 45.4	0.997	0–40	34.66 ± 1.61
4	isoorientin	6.448	447.0	327.0*	35	y = 545.5x- 45.4	0.997	0–40	34.66 ± 1.35
5	*p*-coumaric acid	7.462	163.0	119.0*	33	y = 427.7x + 296.2	0.996	0–20	26.69 ± 0.92
6	vitexin	9.477	431.0	311.2*	20	y = 551.5x + 64.3	0.999	0–20	148.42 ± 7.19
7	fumalic acid	9.612	193.1	134.2*	20	y = 28544.4x- 90877.6	0.983	4–8	265.03 ± 12.56

^1^Data was shown as mean ± SD (n = 5). *Quantitation.

### Animal Treatment and Designation

A total of 800 1-day-old Arbor Acres broilers (local commercial company) were randomly assigned into 4 groups (8 pens with 25 birds per pen): birds supplemented with a basal diet without any additions (Control), birds supplemented with a basal diet with the addition of 20 mg bacitracin/kg (Anti), birds supplemented with a basal diet with the addition of 50 mg BLF/kg (BlfL), and birds supplemented with a basal diet with the addition of 250 mg BLF/kg (BlfH). The birds were reared on the floor and had free access to feed and water for the experiment’s 21-day duration. The room temperature was 35°C at the beginning of the trial and decreased by 2°C per week. The composition and nutritional content of the basal diet are listed in Table 2.

**TABLE 2:** Composition and nutrient levels of the basal experimental diet (air-dry basis).

Ingredients	Content (%)
corn	56.33
soybean meal	24.50
fish meal	5.00
limestone	1.30
soybean oil	1.20
corn gluten meal	2.00
fermented soybean meal	1.67
vitamin-mineral premix^1^	3.00
Total	100.00
nutrient levels	DM (%)
AME (kcal/kg)^2^	2,949
crude protein	20.60
crude fat	4.90
lysine	1.17
methionine + cysteine	1.45
threonine	0.87
tyrosine	0.26
calcium	1.00
available P	0.40

*^1^Supplied per kilogram of diet: vitamin A (retinyl acetate), 1,500 IU; cholecalciferol, 200 IU; vitamin E (DL-α-tocopheryl acetate), 10 IU; riboflavin, 3.5 mg; pantothenic acid, 10 mg; niacin, 30 mg; cobalamin, 10 μg; choline chloride, 1,000 mg; biotin, 0.15 mg; folic acid, 0.5 mg; thiamine, 1.5 mg; pyridoxine, 3.0 mg; Fe, 80 mg; Zn, 40 mg; Mn, 60 mg; I, 0.18 mg; Cu, 8 mg; Se, 0.15 mg. ^2^AME, apparent metabolizable energy; DM, dry matter.*

At the end of the trial, 32 birds (1 bird per pen, 8 birds per treatment group) were randomly selected and slaughtered for sampling. Blood was collected using a coagulation-promoting tube, and allowed to stand at 28°C for 5 h. After The serum samples were obtained by centrifugation at 3,000 *g* for 15 min, and stored under −80°C prior to the detection of antioxidant capacity, immune parameters, and the non-targetted metabolome. The caecal contents were gathered into an aseptic cryopreservation tube and stored under −80°C prior to the detection of short-chain fatty acids (SCFAs) and microbial communities. The intestinal content was gently washed using sterile phosphate-buffered saline (PBS), and jejunal and ileal mucosal samples were gathered using sterile slides, then stored under −80°C prior to cytokine detection.

### Antioxidant Indices

The serum concentration of alanine aminotransferase (ALT), aspartate aminotransferase (AST), antioxidant capacity (T-AOC), malondialdehyde (MDA), superoxide dismutase (SOD), and glutathione peroxidase (GSH-Px) were tested with commercial kits from Nanjing Jiancheng Bioengineering Institute (Nanjing, China).

### Immune Parameters

The serum and mucosal cytokines, including interleukin-6 (IL-6), interleukin-8 (IL-8), and interleukin-1β (IL-1β), as well as the serum immunoglobulins (immunoglobulin A, IgA; immunoglobulin M, IgM; immunoglobulin Y, IgY), were measured by specific ELISA kits (Cusabio, Wuhan, China), following the manufacturer’s instructions.

### 16S rRNA Sequencing

The caecal contents of two replicate birds per pen were mixed into one biological sample. The genomic bacterial DNA of the caecal content was extracted using a DNeasy PowerSoil Kit (QIAGEN Sciences, Inc., Germantown, MD, United States), following the manufacturer’s instructions. After performing quality and quantity assessments, PCR amplification of 16S rRNA genes employing diluted DNA as the template was conducted to perform. Then, the V3–V4 variable regions were amplified for the analysis of the microbial community using the specific primers 343F (5′-TACGGRAGGCAGCAG-3′) and 798R (5′-AGGGTATCTAATCCT-3′). Sequencing was performed using the Illumina MiSeq platform.

The QIIME software (version 1.8.0) was used to decrease the noise of the sequences and remove the chimaeric reads. The operational taxonomic unit (OTU) cluster was generated from clean reads utilising Vsearch software (version 2.4.2) with 97% similarity. The degree of caecal microbial diversity within each treatment group was described by α-diversity analysis (chao_1, shannon, and observed_species); and the differences among all the treatment groups were used for describing the β-diversity analysis (principal component analysis, PCA; principal coordinate analysis, PCoA). Furthermore, the composition of known microbial gene functions was predicted by the phylogenetic investigation of communities by reconstruction of unobserved states (PICRUSt) software with data obtained from the Greengenes database, which were subjected to a Kruskal–Wallis test to assess the differences among the samples.

### UHPLC-Q-TOF/MS for Serum Metabolome Analysis

Serum samples from 32 broilers (eight birds per treatment group) were analysed to check for metabolite changes; the non-targetted serum metabolome was analysed using the method described in Wang et al. (15). The serum extract was chromatographically separated by an Agilent 6545 Q-TOF/MS system (Agilent Technologies). All quality control samples were mixed injected at the same time, which each sample was 5 μL. Additionally, the UHPLC system was equipped with an Agilent Zorbox Eclipse Plus C18 (2.1 mm × 100 mm, 1.8 μm; Waters Corp., Milford, MA, United States). Mobile phases A and B consisted of 10 mM ammonium formate and 1 μL formic acid (95% ACN) and 10 mM ammonium formate and 1 μL formic acid (50% ACN), respectively. In the negative ionisation mode, mobile phase A consisted of 10 mM ammonium acetate with a pH-value 9.0 utilising an ammonium hydroxide solution (95% ACN) and mobile phase B (pH 9.0) consisted of 10 mM ammonium acetate (50% ACN). The solvent gradient elution involved working in a positive ionization mode in the following sequence: 0-2 min, 5% B; 2-20.0 min 5-100% B; 20-25 min, 100% B. A 2 μL sample was injected, and the flow rate was set to 0.3 mL min^–1^.

The UHPLC system connecting an Agilent 6545 ESI-Q-TOF high-resolution accurate-mass spectrometer (Agilent Technologies). The modes of positive and negative ionisation were both employed in present study. The MS conditions were set as follows: capillary voltage, 3.5 kV for the positive mode; gas temperature, 325°C; drying gas flow rate, 11 L min^–1^; nebulizer pressure, 241.32 kPa; sheath gas temperature, 370°C; sheath gas flow rate, 11 L min^–1^; mass range, 100–1,700 m/z. Finally, the metabolites were identified with the Agilent MassHunter Profinder software (Agilent Technologies) and the METLIN database.

### Caecal SCFA Analysis

The caecal SCFA concentrations were quantified by gas chromatography according to the methodology described in our previous study (16). Briefly, 1 g of caecal digesta was blended with 6% phosphorous acid (m/v, 1:4), and the supernatant fluid was collected after vibration and centrifugation. Then, the external standards of SCFAs, comprising acetic acid, propionic acid, butyrate, isobutyric acid, isovalerate, and valerate were purchased from Sigma-Aldrich (Shanghai, China). The standards and samples were injected into and run through an Agilent Technologies 7890B GC System and a flame ionization.

### Statistical Analysis

Significant differences between and within all treatment groups were analysed in IBM SPSS Statistics for Windows, version 21 (IBM Corp., Armonk, NY, United States) one-way ANOVA with using Tukey’s test. A *p* value < 0.05 was regarded as statistically significant; 0.05 < *p* < 0.1 was regarded as a tendency for change, but not a significant difference. The non-targeted serum metabolome was analyzed using the KEGG database, SIMCA-P version 13.0 (Sartorius Stedim Biotech Ltd., Umea, Sweden), and MultiExperiment Viewer version 4.8 (Quantitative Biomedical Research Center, Boston, MA, United States), consisting of PCA, OPLS-DA, and a heat map. The metabolomic data used in the analysis for distinguishing pathways were those that exhibited a fold change >2 and had a *p* value < 0.05, as determined by a *t*-test. MetaboAnalyst 4.0^1^ and KEGG^2^ were used to analyse the metabolic pathways, based on the significantly changed serum metabolites (*p* < 0.05). Diagrams of the data were produced in GraphPad Prism 6.0 (GraphPad Software Inc., San Diego, CA, United States).

## Results

### Major Composition of BLF

As is shown in Table 1, fumaric acid (26.5 mg kg^–1^) and vitexin (14.8 mg kg^–1^) were the predominant BLF components identified in the present study, as well as orientin (3.7 mg kg^–1^), isoorientin (3.7 mg kg^–1^), *p*-coumaric acid (2.7 mg kg^–1^), and chlorogenic acid (2.1 mg kg^–1^).

### Effects of Dietary BLF on Growth Performance

**FIGURE 1:**
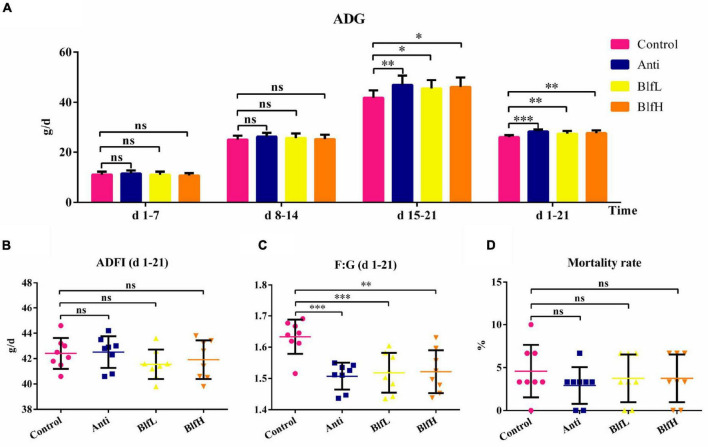
Effects of Anti, BlfL, and BlfH on **(A)** ADG, **(B)** ADFI, **(C)** F:G, and **(D)** mortality rate in broilers from day 1–21. *p < 0.05, ^**^p < 0.01, ^***^p < 0.001, compared to the control treatment (*n* = 8).

Figure 1A showed that both the BLF supplementation and antibiotics significantly improved (*p* < 0.05) the average daily gain (ADG) of birds compared to the control treatment from days 15–21 and days 1–21, while no significant ADG differences were detected in any of the treatment groups from days 1–7 and 8–14. Moreover, BLF- and antibiotic-supplemented birds had lower (*p* < 0.01) feed/gain (F:G) ratios compared to Control birds (Figure 1C). Additionally, there were no significant differences in the average daily feed intake (ADFI) and mortality rate of birds among the treatment groups (Figures 1B,D).

### Effects of Dietary BLF on Serum Antioxidant Capacity

**FIGURE 2:**
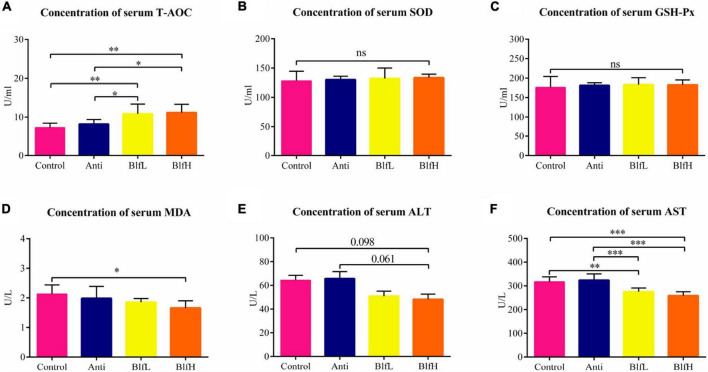
Effects of Anti, BlfL, and BlfH on the serum contents of **(A)** T-AOC, **(B)** SOD, **(C)** GSH-Px, **(D)** MDA, **(E)** ALT, and **(F)** AST in broilers on day 21. *p < 0.05, ^**^p < 0.01, ^***^p < 0.001, tendency changes but no significant difference were considered at 0.1 < *p* < 0.05, compared to the control treatment (*n* = 8).

Both BlfL and BlfH birds had higher (*p* < 0.05) T-AOC serum contents in comparison with Control and Anti birds (Figure 2A). There were no significant differences among the SOD and GSH-Px contents of the different bird groups (Figures 2B,C). The BlfH supplementation significantly decreased the MDA concentration compared to the control treatment (Figure 2D). Compared to the control treatment, the dietary BLF decreased the ALT content, however the difference was not significant (Figure 2E). Moreover, neither the BlfL nor the BlfH birds had significantly higher AST serum concentrations than the Control or Anti birds (Figure 2F).

### Effects of Dietary BLF on Immune Response

**FIGURE 3:**
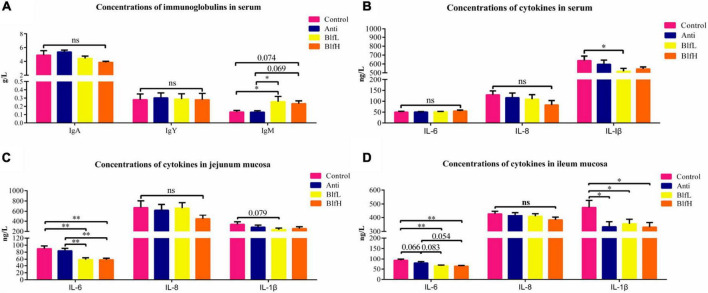
Effects of Anti, BlfL, and BlfH on **(A)** concentrations of serum immunoglobulins, **(B)** concentrations of serum cytokines, **(C)** concentrations of jejunum mucosal cytokines and **(D)** concentrations of ileum mucosal cytokines in broilers on d 21. *p < 0.05, ^**^p < 0.01, tendency changes but no significant difference were considered at 0.1 < *p* < 0.05, compared to the control treatment (*n* = 8).

BlfL birds had higher (*p* < 0.05) serum IgY contents than Control and Anti birds; however, there were no substantial differences among the IgA and IgM values of all birds (Figure 3A). Compared to the control and antibiotic treatments, the BlfL supplementation decreased the serum IL-1β activity of birds, while there were no significant differences between IL-6 and IL-8 (Figure 3B). Dietary BLF significantly decreased (*p* < 0.01) the jejunal mucosal IL-6 content compared with the control or antibiotic treatments (Figure 3C), but there were no significant differences among the IL-8 values of all birds. Compared with the control treatment, BLF supplementation significantly decreased (*p* < 0.05) the ileal mucosal IL-6 content (Figure 3D). No remarkable differences were detected among the IL-8 contents of all birds. Compared to the control treatment, both the BLF and antibiotic treatments significantly decreased (*p* < 0.05) the ileal mucosal IL-1β activity of birds.

### Effects of Dietary BLF on Caecal Microflora

**FIGURE 4:**
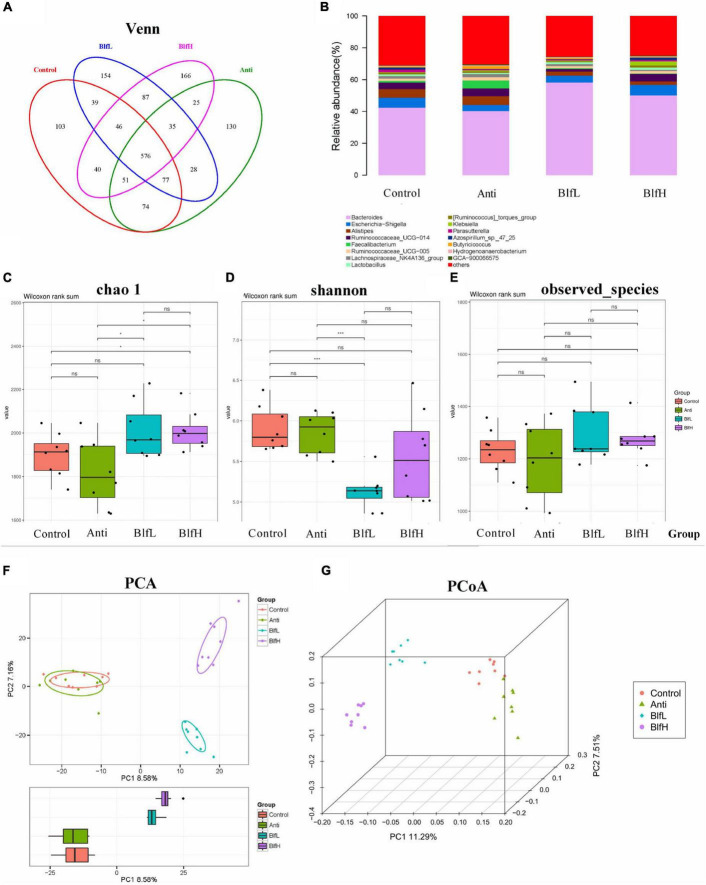
Effects of Anti, BlfL, and BlfH on cecal microbial community in broilers contents on day 21. **(A)** Venn diagram. **(B)** Top 15 cecal microflora genera. **(C)** Chao_1 value. **(D)** Shannon value. **(E)** observed_species. **(F)** PCA plot. **(G)** PCoA plot. *p < 0.05, ***p < 0.001, tendency changes but no significant difference were considered at 0.1 < *p* < 0.05, compared to the control treatment (*n* = 8).

All birds shared 576 OTUs of caecal microbiota, as exemplified by a Venn diagram; among these, the Control, Anti, BlfL, and BlfH birds had 103, 130, 154, and 166 OTUs, respectively (Figure 4A). Out of the top 15 caecal microflora genera, we found that *Bacteroides*, *Escherichia-Shigella*, *Alistipes*, Ruminococcaceae UCG-014, *Faecalibacterium*, Ruminococcaceae UCG-005, Lachnospiraceae NK4A136 group, *Lactobacillus*, and *Klebsiella* predominated the caecal microflora of all birds (Figure 4B). The alpha diversity analysis indicated that BlfH birds had a higher chao_1 index than Control birds, with both BlfL and BlfH birds having higher chao_1 indices than Anti birds (Figure 4C). The shannon parameter decreased more under the BlfL than under the control and antibiotic treatments (Figure 4D). The observed_species parameter did not differ significantly among the birds (Figure 4E). The PCA plot indicated that the BLF samples were separated from the Control and Anti samples (Figure 4F), whereas the samples of each treatment were well separated from those of other groups in the PCoA three-dimensional plot (Figure 4G).

**FIGURE 5:**
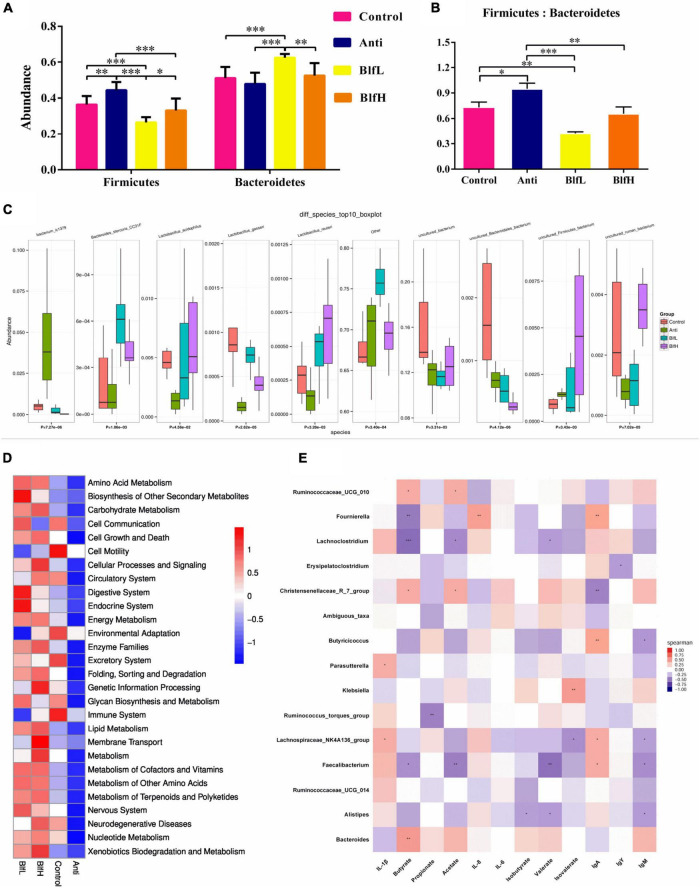
Summary of cecal microbial community in broilers contents on day 21. **(A)** The relative abundance of Firmicutes and Bacteroidetes. **(B)** Firmicutes/Bacteroidetes. **(C)** Kruskal–wallis analysis. **(D)** PICRUSt analysis. **(E)** The correlation between cecal SCFAs, immune parameters and the top 15 distinguished cecal bacteria strains in genus level between BLF treatments and control group. *p < 0.05, ^**^p < 0.01, ^***^p < 0.001, compared to the control treatment (*n* = 8).

Compared with the Control birds, Anti (*p* < 0.01) and BlfL (*p* < 0.001) birds had higher and lower relative abundances, respectively, of Firmicutes (Figure 5A). BLF-supplemented birds had a higher relative abundance of Firmicutes than Anti birds (*p* < 0.001), while BlfH birds had a higher (*p* < 0.05) Firmicute abundance than BlfL birds. Additionally, the BlfL birds had a higher relative abundance of Bacteroidetes than Control and Anti birds (*p* < 0.001). Moreover, BlfL (*p* < 0.01) and Anti birds (*p* < 0.05) had higher and lower Firmicutes:Bacteroidetes ratios, respectively, than Control birds. Both BlfL and BlfH birds had lower (*p* < 0.01) Firmicutes:Bacteroidetes ratios than Anti birds (Figure 5B). Compared to the Control and Anti supplementation, BLF supplementation increased (*p* < 0.05) the *Bacteroides stercoris* CC31F and *Lactobacillus reuteri* concentrations (Figure 5C). In addition, BLF-fed birds had a higher (*p* < 0.05) relative abundance of *Lactobacillus acidophilus* and *Lactobacillus gasseri* than Anti birds. The antibiotic-supplemented birds had higher Bacterium ic1379 than birds subjected to the other treatments. The PICRUSt analysis revealed that higher heatmap scores of known functional genes involved in the metabolism of amino acids, carbohydrates, vitamins, energy, lipids, nucleotides, terpenoids, polyketides, and others, as well as in cellular processes and signalling, enzyme families, and xenobiotic biodegradation and metabolism were induced by the orally BLF supplementation resulted than the control and antibiotic treatments (Figure 5D).

Furthermore, a Spearman’s rank correlation of the 15 top distinguished bacterial genera, serum immunoglobulins, ileal mucosal cytokines, and caecal SCFAs (Figure 5E) revealed that the butyrate content was positively correlated with *Bacteroides*, Rumicococceae UCG 010, and Christensenellaceae R-7 group and negatively correlated with *Fournierella*, *Lachnoclostridium*, and *Faecalibacterium*. The acetate concentration was positively correlated with Rumicococceae UCG 010 and Christensenellaceae R-7 group and negatively correlated with *Lachnoclostridium* and *Faecalibacterium*. There were positive correlations between valerate and *Alistipes*, *Lachnoclostridium*, and *Faecalibacterium*. The serum IgA content was positively correlated with *Fournierella*, *Butyricicoccus*, Lachnospiraceae NK4A136 group, and *Faecalibacterium* and negatively correlated with *Christensenellaceae* R-7 group. The serum IgM level was negatively correlated with *Alistipes*, Lachnospiraceae NK4A136 group, *Butyricicoccus*, and *Faecalibacterium*. The ileal mucosal IL-1β content was positively correlated with *Parasutterella* and Lachnospiraceae NK4A136 group.

### Effects of BLF on the Serum Metabolome

**FIGURE 6:**
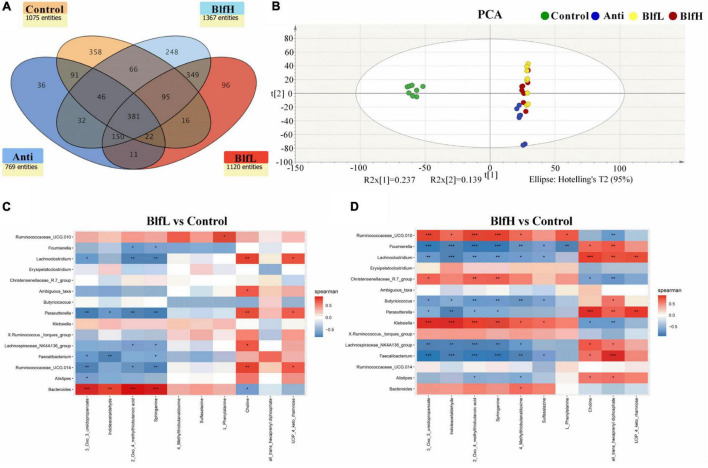
**(A)** Venn diagram exhibiting the common and particularly observed entities in serum non-targeted metabolome of broilers on day 21. **(B)** PCA plot of broilers’ serum metabolome. Panels **(C,D)** represent the correlation between significant changed serum metabolites and the top 15 distinguished cecal bacteria strains in genus level between BLF treatments and control group. *p < 0.05, **p < 0.01, ***p < 0.001, compared to the control treatment (*n* = 8).

In terms of the serum metabolome, all birds shared 381 metabolites, with Control, Anti, BlfL, and BlfH birds having 358, 36, 96, and 248 metabolites, respectively (Figure 6A). Evidently, the BLF supplementation increased the serum metabolites compared with the control and antibiotic treatments, with BlfL and BlfH birds having 1,120 and 1,367 metabolites, respectively. The PCA plot revealed that the BLF-treated birds were well separated from the Control birds; among the former, the BlfL and BlfH samples were closer to each other (Figure 6B). Specifically, 22 significantly changed serum metabolites (up-regulated or down-regulated) were checked within all the treatment groups, and screened for a log2 fold change > 2 and a *p*-value < 0.05 (Table 3). Compared with Control birds, the BLF-treated birds had remarkably up-regulated serum concentrations of indoleacetaldehyde, sphinganine, and phenanthrene-4,5-dicarboxylate (mainly related to amino acid and sphingolipid metabolism), and down-regulated serum choline, hypaconitine, and acetyltropine, related to amino acid and alkaloid metabolism. Moreover, compared to the control treatment, the BlfH treatment resulted in up-regulated (*p* < 0.05) serum concentrations of L-phenylalanine, L-homoserine lactone, slaframine, and 4-methylthiobutanaldoxime (mainly related to amino acid, lipid, and alkaloid metabolism), and down-regulated all-trans-hexaprenyl diphosphates, related to terpenoid metabolism in birds. In addition, the BlfH supplementation significantly up-regulated the small peptide contents (Lys-Gly-Val, Thr-Leu-Pro, and Arg-Pro-Gly) compared to the control treatment. The dietary supplementation with BLF and antibiotics severely decreased the concentration of *N*-acetylgalactosaminyl lactose, UDP-4-keto-rhamnose, gymnodimine, and glycidyl oleate.

**TABLE 3:** Comparison of the transportation of twenty-two metabolites across the broiler serum^1^.

Compounds	Formula	Related Category	RT (min)	mass (m/z)	Ion Species	BlfL VS Control		BlfH VS Control		Anti VS Control	
						trend	*p* value	trend	*p* value	trend	*p* value
Choline	C5 H14 N O	Amino acids	1.114	103.099	[M + H] + [M + Na] +	down^2^	0.000	down	0.000	/^4^	0.146
Indoleacetaldehyde	C10 H9 N O	Amino acids	3.026	159.068	[M + H] +	up	0.000	up^3^	0.000	/	0.816
L-Phenylalanine	C9 H11 N O2	Amino acids	9.618	165.079	[M + H] +	/	0.993	up	0.004	/	0.801
2-Oxo-4-methylthiobutanoic acid	C16 H10 O4	Amino acids	16.560	148.017	[M + H] +	up	0.000	up	0.000	up	0.000
L-Homoserine lactone	C4 H7 N O2	Lipids	1.443	101.049	[2M + H] + [2M + Na] + [M + H] +	/	0.107	up	0.000	/	0.864
Sphinganine	C18 H39 N O2	Lipids	12.471	301.296	[M + H] +	up	0.000	up	0.000	up	0.000
Slaframine	C10 H18 N2 O2	Alkaloids	5.315	198.136	[M + H] +	/	0.177	up	0.000	/	0.013
Hypaconitine	C33 H45 N O10	Alkaloids	7.796	632.339	[M + H] +	down	0.003	down	0.004	down	0.031
Acetyltropine	C10 H17 N O2	Alkaloids	13.439	183.126	[M + H] +	down	0.000	down	0.000	/	0.678
all-trans-hexaprenyl diphosphate	C30 H52 O7 P2	Terpenoids	6.709	586.318	[M + H] + [M + K] + [M + Na] +	/	0.169	down	0.000	/	0.386
Lys Gly Val	C13 H26 N4 O4	Peptides	1.847	324.178	[M + H] +	up	0.027	up	0.001	up	0.088
Thr Leu Pro	C15 H27 N3 O5	Peptides	5.191	329.195	[M + H] +	/	0.993	up	0.005	/	0.819
Arg Pro Gly	C13 H24 N6 O4	Peptides	8.354	328.185	[M + H] +	/	0.112	up	0.000	/	0.852
Sulfasalazine	C18 H14 N4 O5 S	Others aminosalicylic acid	1.755	398.07	[M + 2H] + 2[M + H] +	/	0.176	up	0.026	/	0.868
3-Oxo-3-ureidopropanoate	C4 H6 N2 O4	Others pyrimidine	1.764	146.035	[2M + H] + [M + H] +	up	0.000	up	0.000	/	0.396
4-(Methylnitrosamino)-1-(3-pyridyl)-1-butanol	C10 H15 N3 O2	Others xenobiotics	2.048	226.139	[M + H] +	down	0.033	down	0.000	/	0.931
4-Methylthiobutanaldoxime	C5 H11 N O S	Others glucosinolate	3.405	133.056	[M + H] +	/	0.352	up	0.000	/	0.836
Phenanthrene-4,5-dicarboxylate	C16 H10 O4	Others polycyclic aromatic hydrocarbon	4.375	288.042	[M + H] +	up	0.000	up	0.000	up	0.000
*N*-Acetylgalactosaminyl lactose	C20 H35 N O16	Others enzyme	14.078	567.182	[M + H] +	down	0.000	down	0.000	down	0.000
UDP-4-keto-rhamnose	C15 H22 N2 O16 P2	Others amino and nucleotide sugar	14.429	548.046	[M + H] +	down	0.000	down	0.000	down	0.000
Gymnodimine	C32 H45 N O4	Others marine biotoxins	16.782	507.336	[M + H] + [M + Na] +	down	0.000	down	0.000	down	0.000
Glycidyl oleate	C21 H38 O3	Others carcinogens	19.281	338.283	[M + H] +	down	0.000	down	0.000	down	0.000

*^1^Control: basal diet; BlfL: basal diet + 50 mg BLF/kg; BlfH: basal diet + 250 mg BLF/kg; Anti: basal diet + 20 mg antibiotic/kg. ^2^The distinguished metabolite was down-regulated in the BlfL group compared to the Control group. ^3^The distinguished metabolite was up-regulated in the BlfH group compared to the Control group. ^4^No statistical difference between the Anti group and Control group.*

The metabolomic data analysis with MetaboAnalyst 4.0 using *Gallus gallus domesticus* specific metabolic pathways, revealed that, compared to the control treatment, the dietary BlfL up-regulated the serum sphinganine, related to sphingolipids, and indole-3-acetaldehyde retinol, related to tryptophan metabolism, while it down-regulated the serum choline, related to glycerophospholipid metabolism, and 4-methylthio-2-oxobutanoic acid, related to cysteine and methionine metabolism (Table 4). In addition, compared to the control treatment, the dietary BlfH up-regulated the serum L-phenylalanine, which is associated with phenylalanine, tyrosine, and tryptophan biosynthesis. Therefore, it could be suggested that sphinganine, indole-3-acetaldehyde retinol, choline, 4-methylthio-2-oxobutanoic acid, and L-phenylalanine are the serum biomarkers in broilers supplemented with BLF.

**TABLE 4:** Metabolic pathway and biomarkers with significant differences between BLF and Control groups^1^.

Related metabolism pathway	Biomarkers	Hits	Total	Impact	BlfL VS Control			BlfH VS Control		
					trend	*p*-Value	-log10(p)	Trend	*p*-Value	-log10(*p*)
Glycerophospholipid metabolism	Choline	1	35	0.03	Down^2^	0.000	21.4	Down	0.000	19.9
Cysteine and methionine metabolism	4-Methylthio-2-oxobutanoic acid	1	33	0.06	Down	0.000	19.1	Down	0.000	18.0
Sphingolipid metabolism	Sphinganine	1	21	0.15	Up	0.000	17.3	Up^3^	0.000	16.3
Tryptophan metabolism	Indole-3-acetaldehyde	1	39	0.01	Up	0.000	4.97	Up	0.000	14.9
Phenylalanine, tyrosine and tryptophan biosynthesis	L-Phenylalanine	1	4	0.50	/^4^	0.957	0.02	Up	0.041	1.39

*^1^Control: basal diet; BlfL: basal diet + 50 mg BLF/kg; BlfH: basal diet + 250 mg BLF/kg; Anti: basal diet + 20 mg antibiotic/kg. ^2^The distinguished metabolite was down-regulated in the BlfL group compared to the Control group. ^3^The distinguished metabolite was up-regulated in the BlfH group compared to the Control group. ^4^No statistical difference between the Anti group and Control group.*

### Correlation Between Caecal Microflora and Identified Serum Metabolites

In the present study, we analysed the correlation between caecal microflora and serum metabolome changes, for which we selected the top 15 distinguished bacterial strains at the genus level and the significantly changed serum metabolites (Figures 6C,D). The data obtained from the BlfH and Control groups revealed that Ruminococcaceae UCG.010, Christensenellaceae R-7 group, *Klebsiella*, *Bacteroides*, and *Ruminococcus* torques group were positively correlated with the serum 3-oxo-3-ureidopropanoate, indoleacetaldehyde, 2-oxo-4-methylthiobutanoic acid, sphinganine, and 4-methylthiobutanaldoxime concentrations, with the former three bacterial strains showing higher (*p* < 0.05) correlations among all distinguished bacterial strains. In addition, within the negatively correlated bacterial strains, *Fournierella*, *Lachnoclostridium*, *Butyricicoccus*, and *Faecalibacterium* showed higher (*p* < 0.05) correlation with the serum content of 3-oxo-3-ureidopropanoate, indoleacetaldehyde, 2-oxo-4-methylthiobutanoic acid, sphinganine, 4-methylthiobutanaldoxime, and sulfasalazine. Moreover, *Fournierella*, *Lachnoclostridium*, *Parasutterella*, *Lachnospiraceae* NK4A136 group, *Faecalibacterium*, and *Alistipes* were positively correlated with the serum choline and all-trans-hexaprenyl diphosphate.

### Effects of Dietary BLF on Caecal SCFAs

**FIGURE 7:**
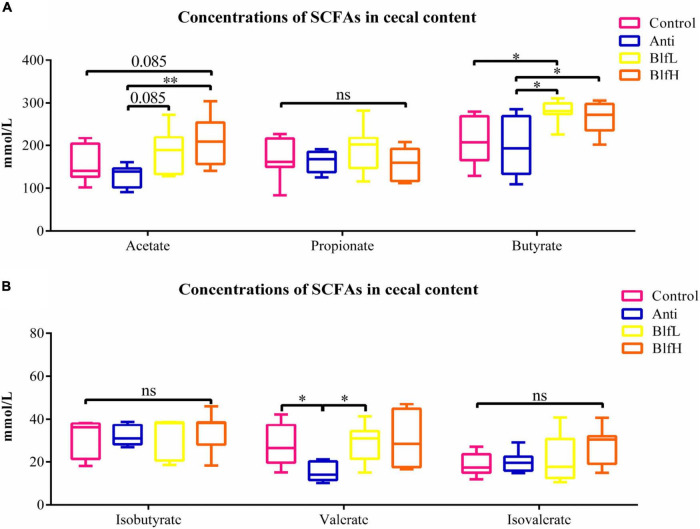
Effects of Anti, BlfL, and BlfH on the cecal levels of **(A)** acetate, propionate, and butyrate, **(B)** isobutyrate, valerate, and isovalerate in broilers on day 21. *p < 0.05, ^**^p < 0.01, tendency changes but no significant difference were considered at 0.1 < *p* < 0.05, compared to the control treatment (*n* = 8).

As is shown in Figure 7A, birds supplemented with BlfH had higher (*p* = 0.085) acetate concentrations in their caecal digesta than Control birds, whereas the BlfL and BlfH birds had higher acetate concentrations than Anti birds (*p* = 0.085, *p* < 0.05). Compared with the Anti treatment, the BLF treatment induced higher butyrate concentrations in the caecal digesta of birds. Additionally, BlfH birds had higher butyrate contents compared to Control birds (*p* < 0.05). Compared to the Anti treatment, both the BlfL and control treatments increased the valerate concentrations of the birds (*p* < 0.05, Figure 7B). There were no significant differences among the propionate, isobutyrate, and isovalerate contents of the birds.

## Discussion

### Major Composition of BLF

Previous studies have reported that the major bioactive substances of BLFs are flavonoids, phenolic acids, and coumaric lactones, as well as the compounds orientin, homoorientin, caffeic acid, and others (17–19). In addition, the main biological functions of bamboo leaves are represented by two major types of polyphenols, which consist of C-glycoside flavonoids and phenolic acid (20, 21). The antioxidant capacity of the n-butanol fraction of bamboo leaf was expressed in terms of the highest total phenolic content and total flavonoid content, for instance characteristic flavonoids and phenolic acids (22). As legal and safe additives, the characteristic antioxidants of BLFs are listed in Chinese National Standard GB 30615-2014. These consist of chlorogenic acid, caffeic acid, orientin, isoorientin, *p*-coumaric acid, vitexin, isovitexin, and fumaric acid. The Bamboo (*Dendrocalamus membranaceus*) used in our trial is widely spread in China, which rich of great economic and ecological value. The present study found that this bamboo composition is similar to that of BLF antioxidants in GB 30615. As expected, bamboo varieties and their growth regions will also have significant effects on BLF composition.

### Effects of Dietary BLF on Growth Performance

Studies have shown that BLEs have multiple biological effects, especially on oxidative injury, as well as in terms of protecting the cardiovascular and cerebrovascular systems (6). The BLE treatment significantly increased the ADG and ADFI and decreased the F:G ratio of broilers. Li et al. (23) also reported that dietary BLF at 2.5 g/kg increased the body weight gain of broiler chickens. Research has shown that even under the effect of various stresses, BLF supplementation can counteract growth-suppressing effects in broiler chickens (24). In our study, birds supplemented with BLF had higher ADG than Control birds from days 1–21, and lower F:G ratios compared to the control and antibiotic treatment groups, with no substantial differences between BlfH and BlfL birds; these results are consistent with those of previous studies.

### Effects of Dietary BLF on Serum Antioxidant Capacity

The serum antioxidant parameters reveal the antioxidant ability of hosts, such as T-AOC, SOD, and GSH-Px, which cooperate to dispel excess free radicals (6). The MDA contents are related to the degree of lipid peroxidation and cell injury caused by free radicals (18). Additionally, serum AST and ALT are commonly used as indicators of the liver function of hosts in clinical medicine, with increasing AST and ALT activities indicating the deterioration of liver cells (19). It has also been shown that BLF enhances cell viability and decreases the MDA, AST, and ALT concentrations (19). Previous studies have confirmed that bioflavonoids are potential natural antioxidants that affect the *in vivo* antioxidant defence systems, for instance SOD and GSH-Px (25–27). Zhang reported that BLF enhances the GSH-Px enzyme concentration in the serum of rats (28). In the present study, the dietary BLF increased the serum T-AOC enzyme concentration and decreased the serum MDA activity, even at low doses, thereby indicating increased blood oxidative stability of birds in their early growth stages. Moreover, decreasing levels of serum AST and ALT were discovered in birds supplemented with BLF, especially in those supplemented with higher levels. Bioflavonoids exhibit a structure-oriented antioxidant capacity, in which the –OH group conducts as a hydrogen donor to peroxy radicals and inhibits hydroxyl peroxide formation (29, 30). Previous studies have reported that BLEs have beneficial effects on inflammation and oxidative stress, which are related to the antioxidant effects of the activation of hepatic phase II enzymes or the AKT pathway (31, 32). Therefore, dietary BLF may increase the antioxidative capacity of birds in the early growth stages.

### Effects of Dietary BLF on Immune Response

It is well known that immunoglobulins are used to assess the immune capacity of hosts owing to their important roles in immune functions (33). The results of the present study revealed that compared to the control treatment, the BLF treatment induced an increase in the serum IgM and a decrease in the serum IL-1β, mucosal IL-6, and IL-1β, which indicate enhanced immune responses in the broilers. In fact, the flavonoid-mediated modulation of the inflammatory response has been extensively investigated in both *in vivo* and *in vitro* trials (34–36). Certain types of polyphenol-derived metabolites produced from colonic microbiota not only inhibit dextran sulfate sodium -induced colitis lipid peroxidation and DNA damage in the digestive tract mucosa but also decrease the fundamental cytokines in the inflammatory response, including TNF-α, IL-1β, and IL-8 (37). Shukla et al. also found that supplementing a basal feed with flavonoids improved mucosal immunity by increasing the concentrations of intraepithelial lymphocytes in the duodenum and jejunum of hosts (38). BLFs have been proven to promote DNA and protein synthesis in immune cells and enhance immunity in mice models (39). Based on the aforementioned studies, we hypothesised that the dietary BLF may regulate the immune responses of broilers by enhancing their mucosal and serum immunity.

### Effects of Dietary BLF on Caecal Microflora

It is well known that the microbial communities in the digestive tract play a significant role in promoting the health status and production efficiency of broilers by improving digestion, regulating immune responses, and limiting pathogenic reproduction (40, 41). The caecum is the major fermentative organ in livestock, in which the microbial community plays a key role in feed utilization (42). Utilizing BLFs as feed additives may inhibit the colonisation of pathogens and increase the abundance of beneficial microbiota, which enhance the birds’ immune responses (43).

In the present study, the results of both the alpha and beta diversity analyses demonstrated the modulation of BLF supplementation on the caecal microflora of birds. Yang et al. discovered that dietary supplementation with combinations of essential oils and BLF could be utilised to replace antibiotics by improving the relative abundance of *Lactobacillus* and *Bifidobacterium* in the caeca of broilers (44). Moreover, a study in pigs confirmed that polyphenol-rich cocoa increased the *Faecalibacterium prausnitzii* levels and decreased the *Firmicutes:Bacteriodetes* ratios (45). We obtained similar results in our experiment, as dietary supplementation with BLFs decreased the Firmicutes:Bacteroidetes ratio, while it increased the relative abundance of *Lactobacillus* and *Bacteroides stercoris* CC31F. Shu et al. also found that BLF supplementation induced changes in the gut microbial community structure of broilers, thereby resulting in greater *Lactobacillus*, *Ruminococcus*, and Lachnospiraceae concentrations (43). Many researchers have found that there is a close relationship among intestinal microflora, SCFAs, and immune responses (46). The Spearman’s rank correlation analysis employed in this study also showed that certain types of caecal microbes, such as *Bacteroides*, Rumicococceae UCG 010, Christensenellaceae R-7 group, *Fournierella*, *Lachnoclostridium*, *Faecalibacterium*, and *Alistipes*, were either positively or negatively correlated with caecal SCFAs or immune parameters in birds. Previous studies also revealed that SCFA-producing bacterial genera, including *Ruminococcus*, *Faecalibacterium*, and Lachnospiraceae, increase the total intestinal SCFAs, acetate, and butyrate, which corroborate our results (23, 47).

### Effects of BLF on the Serum Metabolome

Studies on the role of the intestinal microbial community in the digestion and absorption of nutrients, including lipids, carbohydrates, and proteins, have been conducted using different animal models (48). The results of this study demonstrated the modulation effect of BLF on the caecal microflora of broilers. Additionally, the PICRUSt analysis of caecal 16S rRNA sequencing indicated that the dietary BLF treatment induced higher expression of known functional genes involved in lipid, amino acid, carbohydrate, cofactor, vitamin, terpenoid, and polyketide metabolism. Furthermore, the non-targeted serum metabolome revealed that twenty-two significantly changed serum metabolites were mainly correlated with the metabolism of lipids, amino acids, alkaloids, and others; these results are consistent with the data obtained from the PICRUSt analysis of caecal microbial sequencing. Additionally, the changed serum biomarkers were related to the metabolism of glycerophospholipids, sphingolipids, tryptophan, cysteine, and methionine, as well as the biosynthesis of phenylalanine, tyrosine, and tryptophan in birds supplemented with BLF. Studies have confirmed that glycerophospholipids play an important role in regulating host inflammation and immunity (49, 50), thereby corroborating the BLF immunoregulation capability results obtained in the present study. Additionally, the involvement of sphingolipids in innate immunity against infection from intestinal pathogenic microorganisms has also been confirmed (51), which provides evidence for the BLF-induced microbial modulation demonstrated in this study.

### Correlation Between Caecal Microflora and Identified Serum Metabolites

Studies have confirmed that dietary flavonoids cannot be completely absorbed by the digestive tract and are metabolised by intestinal microflora, which reaffirms that they and their metabolites may play an important role in maintaining the health of the intestinal microbiome (52). However, studies on the relationship between microbial communities and serum metabolites in broilers supplemented with BLF are rare. In the present study, the combined data obtained from the BlfL and Control groups showed that, among all distinguished bacterial strains, *Fournierella*, *Lachnoclostridium*, *Parasutterella*, *Klebsiella*, Lachnospiraceae NK4A136 group, *Faecalibacterium*, and *Bacteroides* played a crucial role in altering the serum metabolites in the present study. Moreover, choline, sphinganine, and *L*-phenylalanine were most affected by the microflora changes based on the metabolite biomarker matched pathways and the correlation between distinguished caecal microflora and serum metabolites that underwent major changes.

### Effects of Dietary BLF on Caecal SCFAs

It is well known that SCFAs that are produced mainly by microbial fermentation can provide important nutrients for the regeneration of intestinal epithelial cells, inhibit the growth of pathogens by reducing intestinal pH values, and indirectly modulate the intestinal microflora (53, 54). The results of the present study showed that the dietary supplementation with BLF induced an increase in the caecal acetate and butyrate contents. In fact, numerous *in vivo* and *in vitro* studies have confirmed that different *Lactobacillus* spp. strains lead to the accumulation of SCFAs, such as acetic acids and butyrate (55–57). A previous study conducted by Gancarčíková et al. confirmed that the increase in the caecal SCFA concentrations (mainly in the butyric, acetic, and propionic acid concentrations) was induced by *L. reuteri* (58). In the present study, the BLF supplementation increased the relative abundance of *L. acidophilus*, *L. gasseri*, and *L. reuteri* compared to the Anti treatment, as well as the relative abundance of *L. reuteri* compared to the control treatment. Therefore, it suggested that the modulatory effect of BLFs on caecal microbiota changes the caecal SCFA levels.

## Conclusion

Dietary BLF supplementation significantly improved the health status of 1 to 21-day-old *Gallus gallus domesticus* broilers, including an increase in the serum IgM content, a decrease in mucosal IL-6 and IL-1β contents, a increase in caecal *Bacteroides stercoris* CC31F and *L. reuteri* concentrations, as well as a increase in the caecal acetate and butyrate contents. Moreover, choline, sphinganine, and *L*-phenylalanine were most affected by the caecal microflora changes based on the metabolite biomarker related pathways.

## Data Availability Statement

The raw data supporting the conclusions of this article will be made available by the authors, without undue reservation.

## Ethics Statement

The animal study was reviewed and approved by the Ethics Committee of Zhejiang Agricultural and Forestry University.

## Author Contributions

GC and JL: data curation. XZ, YaY, and HW: formal analysis. YuY and ZL: investigation. YZ and YaY: methodology. CY and JL: resources. GC: supervision and writing—original draft. CY and GC: writing—review and editing. All authors have read and agreed to the published version of the manuscript.

## Conflict of Interest

JL, XZ, and YZ were employed by Vegamax Biotechnology Co., Ltd. The remaining authors declare that the research was conducted in the absence of any commercial or financial relationships that could be construed as a potential conflict of interest.

## Publisher’s Note

All claims expressed in this article are solely those of the authors and do not necessarily represent those of their affiliated organizations, or those of the publisher, the editors and the reviewers. Any product that may be evaluated in this article, or claim that may be made by its manufacturer, is not guaranteed or endorsed by the publisher.

## Footnotes

1
http://www.metaboanalyst.ca

2
http://www.kegg.jp
