# Supplementary figures and images for: Effects of Oral Administration of Bamboo (Dendrocalamus membranaceus) Leaf Flavonoids on the Antioxidant Capacity, Caecal Microbiota, and Serum Metabolome of Gallus gallus domesticus
Source: Front Nutr. 2022 Mar 3;9:848532. doi: 10.3389/fnut.2022.848532 (PMC8930276; doi:10.3389/fnut.2022.848532)

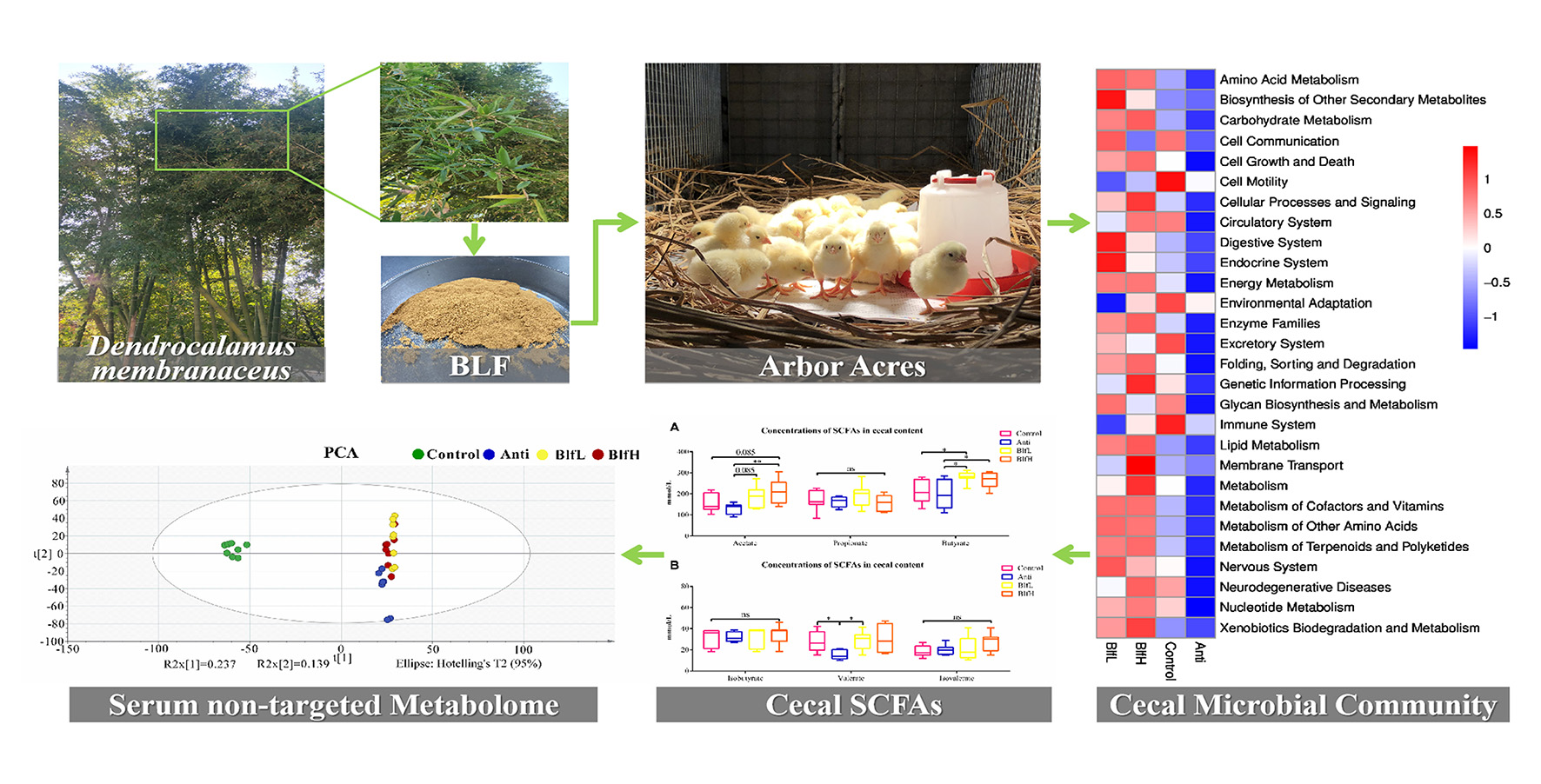

Supplement: Supplementary file 1 [file Image_1.JPEG]
